# Seasonal metabolic oscillation: adiposity cycling as a candidate amplifier of mood seasonality in seasonal affective and bipolar disorders

**DOI:** 10.3389/fpsyt.2026.1921655

**Published:** 2026-08-03

**Authors:** Ece Özlem Öztürk, Ömer Aydemir

**Affiliations:** 1 Department of Psychiatry, Manisa Mental Health and Diseases Hospital, Manisa, Türkiye; 2 Department of Neuroscience, Institute of Health Sciences, Dokuz Eylül University, İzmir, Türkiye; 3 Department of Psychiatry, Faculty of Medicine, Manisa Celal Bayar University, Manisa, Türkiye

**Keywords:** bipolar disorder, circadian clock genes, kynurenine pathway, metabolic psychiatry, mood seasonality, neuroinflammation, obesity, seasonal affective disorder

## Abstract

Seasonal affective disorder (SAD) and bipolar disorder (BD) with a seasonal pattern are conventionally framed as disorders of photoperiod and circadian timing, yet affected individuals also show a striking, reproducible metabolic rhythm—carbohydrate craving and weight gain in winter, appetite reduction and weight loss in summer—that has rarely been integrated into mechanistic models of seasonal mood cycling. Building on an evolutionary “metabolic plasticity” view, in which seasonal shifts in energy storage are conserved adaptations that can become maladaptive in predisposed individuals, we propose the Seasonal Metabolic Oscillation (SMO) hypothesis: that the seasonal expansion and contraction of adipose tissue is not merely a passive symptom but a candidate mediator and amplifier of seasonal mood cycling. We advance two claims of deliberately unequal evidentiary strength. The better-supported core claim is that winter adiposity amplifies and helps maintain the depressive phase, by promoting low-grade neuroinflammation and indoleamine 2,3-dioxygenase (IDO1) activation, which diverts tryptophan into the kynurenine pathway, and by adding leptin resistance and stress-axis dysregulation that together bias the brain toward depression. The more tentative, exploratory claim is that the spontaneous loss of adiposity in summer relieves this inflammatory burden and, through photoperiod-driven changes in dopaminergic and circadian signaling, may lower the threshold for mood elevation in predisposed individuals. Pleiotropic circadian and metabolic variants, including ZBTB20 and NPAS2, are proposed to confer joint vulnerability to SAD, obesity, and bipolar spectrum illness. The model yields a central, falsifiable prediction—that within the same individuals seasonal swings in adiposity statistically precede and account for a defined share of seasonal mood change—and is directly testable through weight-stabilization experimental-medicine trials, within-subject longitudinal biomarker tracking, and Mendelian randomization. We stress that the model’s two central assumptions—that seasonal adiposity change temporally precedes mood change, and that a swing of the modest magnitude typical of SAD is quantitatively sufficient to move central neurochemistry—remain experimentally undemonstrated, and that photoperiod is a plausible common cause of both adiposity and mood that only designs decoupling the two can exclude. If supported, SMO would reframe seasonal mood disorders as recurrent metabolic-neuroimmune-mood conditions and motivate metabolically informed screening, pharmacotherapy, and chronotherapy.

## 1 Introduction

Seasonal affective disorder (SAD) is defined in DSM-5 and ICD-11 not as an independent diagnostic entity but as a “seasonal pattern” specifier applicable to recurrent major depressive disorder (MDD) and to both types of bipolar disorder (BD) (1, 2). The specifier requires that depressive episodes consistently begin in a particular season—almost invariably in autumn or winter—and fully remit at other times, that this cyclical pattern be present for at least two consecutive years, and that seasonal episodes outnumber non-seasonal episodes across the lifetime (3).

Epidemiological data indicate that the global prevalence of SAD is approximately 5%, with prevalence increasing at higher latitudes; for instance, rates have been reported to reach approximately 19–21% in some high northern latitude samples (4, 5). SAD disproportionately affects women, with reported female-to-male ratios ranging from 3.5:1 to 9:1, and the first episode typically occurring around the age of 30 years (6, 7).

The disorder exhibits a marked latitudinal gradient; prevalence varies according to geographic location and diagnostic criteria. In regions such as East Asia, measurement and classification issues may influence prevalence estimates, whereas higher rates have been reported in more extreme northern or southern locations (8, 9). Moreover, clinical SAD should not be conflated with broader seasonal fluctuations in mood and behavior. In a high-latitude population, clinical SAD remained relatively uncommon, whereas seasonality was substantially more prevalent, suggesting a pattern consistent with photoperiod-related regulation of biological rhythms (10).

Epidemiological and clinical data consistently link a seasonal pattern to the bipolar spectrum. A systematic review of 51 studies showed that, in BD, manic and hypomanic episodes exhibit a robust spring-summer peak while depressive episodes cluster in early winter (11). The seasonal pattern (SP) is more prevalent in Bipolar II than in MDD or Bipolar I; a prospective cohort found that Bipolar II carries the highest SAD prevalence among early-onset mood disorders (11). A multicenter study reported that SP is significantly associated with hypomanic features during index depressive episodes, and in a clinical cohort of 708 patients SP was present in 16.5%, with Bipolar II showing a particularly strong association (OR = 2.23, 95% CI 1.40–3.55) (12). Seasonality further maps onto distinct clinical phenotypes and treatment-related outcomes in BD (13). Together, these observations are consistent with—though they do not establish—a shared neurobiological continuum between SAD and bipolar mood cycling (14).

Existing neurobiological models of SAD emphasize two mechanisms: (i) circadian phase disruption, in which prolonged winter melatonin secretion desynchronizes central pacemakers from the photoperiod (15), and (ii) serotonergic dysregulation, characterized by enhanced serotonin-transporter (SERT) activity during winter (16). Both are supported by the antidepressant efficacy of bright-light therapy (17, 18). However, the consistent winter hyperphagia, carbohydrate craving, and weight gain—followed by appetite reduction and weight loss in summer—has not been systematically incorporated into a unifying pathogenetic framework.

**Figure 1:**
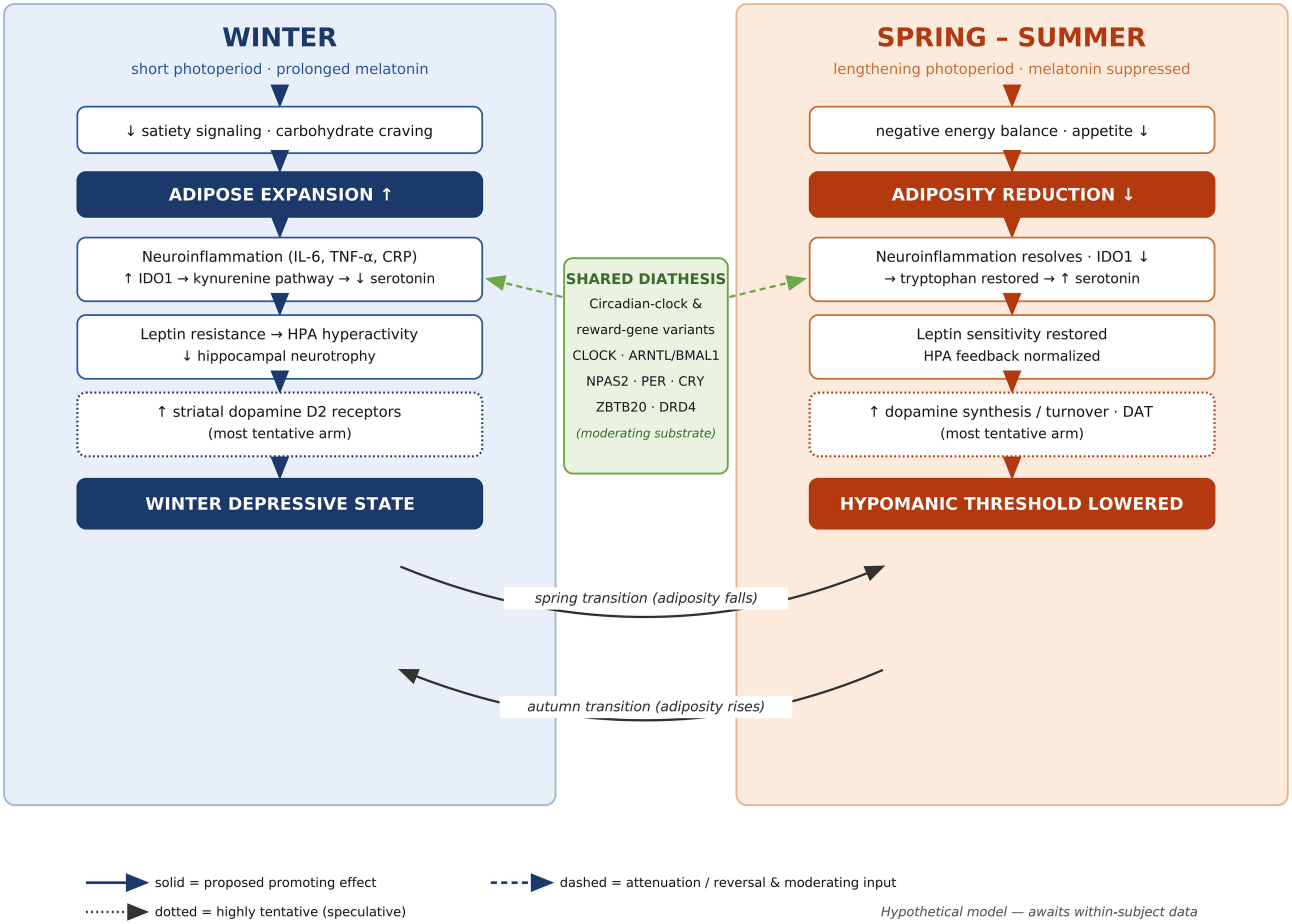
The seasonal metabolic oscillation (SMO) hypothesis. Proposed seasonal loop linking photoperiod, adiposity, and mood polarity. (Left, winter) Short photoperiod and prolonged melatonin secretion reduce satiety signaling and promote carbohydrate-driven hyperphagia and adipose expansion. Increased adiposity drives (i) low-grade neuroinflammation (IL-6, TNF-α, CRP) and IDO1 activation, diverting tryptophan into the kynurenine pathway, lowering serotonin and shifting the pathway toward neurotoxic quinolinic acid and 3-hydroxykynurenine; (ii) leptin resistance with impaired hippocampal neurotrophy and HPA hyperactivity; and (iii) photoperiod-linked changes in striatal dopaminergic signaling (dopamine synthesis/turnover and presynaptic D2/3 autoreceptor regulation; the most tentative arm—note that short winter photoperiods are associated with higher, not lower, striatal D2/3 availability, so this link is shown as parallel to, rather than driven by, the adiposity loop)—together biasing the system toward the winter depressive state. (Right, spring-summer) Lengthening photoperiod restores circadian entrainment and suppresses melatonin, while negative energy balance reduces adiposity, reversing the inflammatory-metabolic burden, restoring serotonergic tone and—via photoperiod-driven dopamine synthesis and transporter dynamics rather than receptor up-regulation—lowering the threshold for hypomanic elevation in predisposed individuals. A shared circadian-clock and reward-gene diathesis (e.g., CLOCK, ARNTL/BMAL1, NPAS2, PER, CRY, ZBTB20, DRD4) acts as a moderating substrate. Solid arrows denote proposed promoting effects; dashed arrows denote attenuation/reversal and moderating input. Arrow rendering additionally encodes evidentiary strength: bold lines correspond to links supported by meta-analytic or well-replicated human data, thin lines to single-study or observational human associations, and dotted lines to the highly tentative, as-yet-untested links—most importantly the dopaminergic arm, which should be read as speculative. The model is hypothetical and awaits within-subject longitudinal confirmation.

We propose that seasonal adiposity may function as a candidate mediator and amplifier—rather than a passive correlate—within the SAD-bipolar continuum. In brief (**Figure 1**); winter weight gain is proposed to promote neuroinflammatory, neuroendocrine, and neurotransmitter changes that deepen and maintain depressive states, whereas summer weight loss may reverse these processes and lower the threshold for hypomanic or manic elevation. Throughout, we distinguish two claims of unequal evidentiary strength. The core thesis—that winter adiposity metabolically amplifies and helps maintain the depressive phase—rests on the firmer foundation of meta-analytic and cohort data. The auxiliary, more exploratory thesis—that summer adiposity loss facilitates hypomanic elevation through dopaminergic mechanisms—is supported only by indirect and anecdotal evidence and is flagged at every step as the model’s most tentative arm. A central challenge for any such account—whether seasonal adiposity mediates the photoperiod–mood relationship or is merely a parallel consequence of changing daylength—is addressed explicitly in Sections 5 and 6. Because this common-cause alternative and the untested sufficiency of a modest seasonal weight swing are the two assumptions on which the entire model rests, we foreground them at the outset rather than treating them as afterthoughts: throughout, adiposity is presented as a candidate mediator whose causal status is provisional and awaits the decoupling designs of Section 6. We call this the Seasonal Metabolic Oscillation (SMO) hypothesis. The argument is built on four converging evidence streams—(i) the metabolic phenotype of SAD, (ii) the bidirectional obesity-mood relationship, (iii) neurobiological mechanisms linking adiposity to mood, and (iv) shared circadian genetic architecture—which we marshal in turn before integrating them into a single model, stating its assumptions, and specifying how it could be falsified.

## 2 The metabolic signature of seasonal affective disorder

The vegetative profile of winter-type SAD is distinct from melancholic depression. Rather than insomnia and appetite suppression, affected individuals exhibit hypersomnia, anergia, social withdrawal, and hyperphagia with specific craving for carbohydrate-rich foods. A systematic review of 11 studies found larger evening meals, more frequent night-time snacking, higher rates of binge eating, and greater external and emotional eating relative to comparison groups (19). Caloric and carbohydrate intake normalize during remission, indicating that hyperphagia is state-dependent (20).

Seasonal body-weight variation is a reproducible feature. In a community study of 636 Amish individuals, a greater winter-to-summer weight differential was inversely associated with plasma adiponectin, implicating adipose dysfunction even at subclinical levels of seasonal weight gain (21). Among individuals with SAD, those with greater pre-existing adiposity and more pronounced emotional and restrained eating styles show the largest seasonal weight change, suggesting that pre-existing adipose burden amplifies the metabolic oscillation (20).

Several systems link seasonal light deprivation to hyperphagia. Short photoperiods and prolonged nocturnal melatonin are associated with blunted satiety signaling, enhanced carbohydrate preference, and reduced energy expenditure (2, 22). Circadian misalignment independently increases metabolic-syndrome and obesity risk through disrupted appetite and glucose regulation (23). Serotonergic dysfunction is also central to carbohydrate craving: enhanced winter SERT activity reduces synaptic serotonin (16), and because insulin-stimulated carbohydrate intake facilitates tryptophan transport across the blood-brain barrier, carbohydrate craving may represent partial self-medication aimed at restoring serotonergic tone (24). Paradoxically, chronic carbohydrate overconsumption sustains weight gain and thereby activates inflammatory pathways that further suppress serotonin synthesis.

Genetic evidence reinforces the metabolic dimension. The dopamine D4 receptor variant DRD4-7R was associated with a threefold increase in binge eating in 131 women with SAD (OR = 3.25, 95% CI 1.43–7.41), and among 182 women with SAD, spring-born 7R carriers showed a maximum BMI of 33.7 ± 8.6 versus 26.7 ± 5.4 kg/m² in non-carriers, with an obesity odds ratio of 4.68 (95% CI 1.67–13.07) (25, 26). These findings have been interpreted as a “seasonal thrifty phenotype”—an evolutionary hypothesis, rather than an established mechanism—in which dopaminergic reward circuitry, food intake, and seasonal energy storage are proposed to be biologically intertwined. CLOCK, ARNTL, and NPAS2 polymorphisms have likewise been associated with seasonal variation in appetite and body weight in healthy adults (27).

## 3 The bidirectional obesity–mood relationship

The obesity-depression relationship is bidirectional and epidemiologically robust. A landmark meta-analysis of longitudinal studies showed that baseline obesity predicts future depression (OR ≈ 1.55) and baseline depression predicts future obesity (OR ≈ 1.58) (28). A bias-adjusted meta-analysis of 21 studies confirmed prospective associations in both directions, particularly in young and middle-aged women (29), and in the Nurses’ Health Study of 65,955 women, depression increased 10-year obesity risk (OR ≈ 1.38–1.51) while baseline obesity modestly raised incident depression (OR ≈ 1.10–1.11) (30).

In BD, obesity prevalence is substantially elevated (≈39%), with high BMI independently associated with chronic course, reduced functioning, greater medical comorbidity, and poorer treatment outcomes (31). A US survey of 1,853 patients with Bipolar I linked obesity to greater comorbidity, lower quality of life, and higher emergency utilization (32); and among adolescents with BD, excess weight is associated with substantially worse clinical outcomes, including higher rates of suicide attempts, hospitalization, and comorbid eating pathology (33). In a pediatric Medicaid cohort, pre-existing obesity preceded BD diagnosis in a notable proportion, raising the possibility that metabolic dysregulation contributes to the phenotypic expression of bipolar illness (34). Importantly, DSM-5 mixed features in both bipolar depression and mania are specifically associated with higher rates of obesity and metabolic disturbance (35), consistent with a model in which adipose burden modulates the polarity and complexity of mood episodes.

## 4 Proposed mechanisms

### 4.1 Neuroinflammation and the kynurenine pathway

Visceral adipose expansion generates a chronic low-grade inflammatory state through secretion of IL-6, TNF-α, and CRP. These mediators act on the brain, activating microglia and sustaining hypothalamic and hippocampal neuroinflammation that disrupts synaptic plasticity, impairs neurogenesis, and promotes depressive-like states (36). A key downstream consequence is activation of indoleamine 2,3-dioxygenase (IDO1), which diverts tryptophan from serotonin-melatonin synthesis into the kynurenine pathway (KP). Under chronic inflammation, the KP favors neurotoxic metabolites—quinolinic acid (an NMDA-receptor agonist) and 3-hydroxykynurenine—over neuroprotective kynurenic acid, and shifts in the kynurenine/tryptophan ratio correlate with depressive severity (37, 38).

In individuals with obesity, elevated CRP is associated with reduced plasma tryptophan and more severe depression (38), and a high-fat-diet mouse model reproduced depressive- and anxiety-like behaviors alongside KP imbalance, with behavioral disturbance tracking neurotoxic-metabolite accumulation (39). These data are consistent with—though they do not establish—the interpretation that winter weight gain may actively participate in maintaining depressive episodes through sustained KP activation and serotonin depletion, whereas the spring-summer reversal of adiposity could reduce IDO1-driven tryptophan diversion, attenuate microglial activation, and improve hippocampal plasticity in a manner mechanistically consistent with seasonal remission. We emphasize that this evidence is largely cross-sectional or derived from chronic-obesity and high-fat-diet models; its application to a months-long seasonal amplitude is an inference that the designs in Section 6 are intended to test. A further complication must be acknowledged here. The serotonergic side of this account cannot be attributed to winter adiposity alone, because the seasonal epidemiology of tryptophan availability does not coincide with the winter adiposity peak: longitudinal sampling shows that plasma L-tryptophan and the tryptophan/competing-amino-acid ratio reach their nadir in spring rather than in winter, paralleling the spring excess of violent suicide (40). This argues against a model in which serotonergic depletion is driven solely by winter fat mass, and it favors a more integrated reading: photoperiod exerts a direct, adiposity-independent influence on tryptophan availability, while winter carbohydrate craving and weight gain may represent, in part, a compensatory (self-medication) effort to sustain insulin-facilitated tryptophan transport into the brain rather than a purely pathogenic process. On this view—consistent with the evolutionary metabolic-plasticity perspective developed in Section 5—adiposity-driven inflammation operates alongside, and partly downstream of, photoperiodic control of tryptophan metabolism rather than replacing it.

### 4.2 Photoperiodic neuroendocrine control: the pars tuberalis–tanycyte thyroid switch

A conserved neuroendocrine pathway provides a direct molecular route from daylength to central metabolic state, upstream of and independent of adipose tissue itself. In seasonal mammals, the duration of nocturnal melatonin secretion is decoded by melatonin MT1 receptors on thyrotroph-like cells of the pars tuberalis of the pituitary. Lengthening photoperiod (short-duration melatonin) increases pars tuberalis expression of thyroid-stimulating hormone β-subunit (TSHβ); this locally released TSH acts retrogradely on TSH receptors of ependymal tanycytes lining the third ventricle, where it reciprocally regulates the deiodinase enzymes DIO2 and DIO3. A shift toward DIO2 raises local hypothalamic conversion of thyroxine (T4) to bioactive tri-iodothyronine (T3), whereas short winter photoperiods favor DIO3 and lower local T3. Because hypothalamic T3 availability gates seasonal programs of appetite, energy expenditure, and body-weight set-point, this pars tuberalis–tanycyte axis constitutes a well-established mechanism of seasonal metabolic adaptation in photoperiodic species (41–43). Its translational relevance to human SAD and BD has not been established and should be regarded as an open question rather than a demonstrated mechanism. Nonetheless, it supplies precisely the upstream neuroendocrine bridge the SMO framework requires: a photoperiod-driven, adiposity-independent route by which shortening autumn–winter daylength could initiate the winter metabolic phenotype—reduced energy expenditure, hyperphagia, and adipose expansion—before, and in parallel with, the adiposity-driven inflammatory–kynurenine cascade developed above. Framing the thyroid switch in this way also clarifies how photoperiod can act as a common cause on the metabolic side of the loop (Sections 5–6): seasonal hypothalamic T3 dynamics therefore become a natural additional mediator to sample in the longitudinal designs of Section 6, and a candidate node at which photoperiodic and adiposity-driven influences on central metabolism converge.

### 4.3 Leptin resistance and HPA-axis dysregulation

Leptin, secreted in proportion to fat mass, acts on mood-relevant circuits via receptors in the hippocampus, amygdala, ventral tegmental area, and hypothalamus, promoting neurogenesis and facilitating serotonergic and dopaminergic transmission (44). Under chronic positive energy balance, leptin resistance develops—high circulating leptin with diminished receptor signaling—impairing these functions. Elevated leptin is specifically associated with major depression with atypical features (the subtype most similar to SAD), with the effect strengthening as adiposity increases (45). In diet-induced obese rodents, hypothalamic-pituitary-adrenal (HPA) responsiveness to leptin is impaired, with blunted STAT3 phosphorylation and elevated IL-1β in brainstem regions regulating the stress axis (46), and in women with obesity, dynamic cortisol modeling shows attenuated feedback efficiency with greater fat mass and insulin resistance (47). Winter adiposity may thus create a neuroendocrine state of leptin resistance and HPA hyperactivity that synergizes with inflammatory and serotonergic mechanisms to consolidate depression, with summer adiposity reduction progressively restoring leptin sensitivity and HPA feedback.

### 4.4 Re-evaluating the dopaminergic axis: an exploratory arm (synthesis versus receptor regulation)

This is the most speculative component of the model, and we now present it explicitly as a subordinate, hypothesis-generating conjecture rather than as a parallel mechanistic pathway: it is retained only because it yields a clean, directional imaging prediction worth testing, not because current evidence makes it believable. Chronic obesity is associated with reduced striatal dopamine D2-receptor (D2R) availability (48, 49), which might tempt the extrapolation that summer weight loss up-regulates D2R and precipitates hypomania. This extrapolation is contradicted by the only direct seasonal measurement available: in a [¹¹C]raclopride PET study, longer daylength was associated with lower—not higher—striatal D2/3 availability, so short winter photoperiods correspond to higher receptor binding, the opposite of a simple adiposity-downregulation account, most plausibly reflecting presynaptic autoreceptor feedback against elevated dopamine synthesis in dark months (50). We therefore retract the receptor-rebound formulation. Any dopaminergic contribution to summer mood elevation is better attributed to photoperiod-driven changes in dopamine synthesis and turnover, dopamine-transporter (DAT) function, and melatonin suppression—processes governed by circadian-clock genes—than to adiposity-driven receptor up-regulation (51); increased summer light acting on melanopsin-expressing intrinsically photosensitive retinal ganglion cells (ipRGCs) offers a further, co-acting photic route to mood elevation (52, 53). Under this revised view, adiposity is at most a chronic modulator of dopaminergic tone, not the proximal seasonal switch. The indirect human evidence sometimes cited for an adiposity-loss-to-mania link—a small, uncontrolled ketogenic-diet case series and a single semaglutide-associated case (54, 55)—is weak and heavily confounded, because both interventions exert potent central effects independent of fat loss; these reports are best treated as hypothesis-generating rather than as support. Accordingly, the dopaminergic arm should be regarded as a priority target for direct, longitudinal testing (Section 6), explicitly framed to measure dopamine synthesis, DAT, and seasonal D2/3 availability, rather than as an established mechanism.

### 4.5 Shared circadian and reward genetic architecture

SAD, obesity, and BD plausibly share a circadian genetic substrate. Clock genes—CLOCK, ARNTL (BMAL1), NPAS2, PER2, PER3, CRY1, CRY2, and RORA/RORB—regulate seasonal adaptation, metabolic homeostasis, and neurotransmitter cycling, and variants have been linked to all three domains (56, 57). For SAD, a phenotypically characterized population study found that combinations of CLOCK, PER2, PER3, CRY2, and ZBTB20 variants increase SAD and seasonality risk in a sex-specific manner (58), and the first GWAS of SAD identified the strongest signal at ZBTB20 (59). For BD, NPAS2 was the variant most robustly associated with SP, with additional signals in CRY2, ARNTL, ARNTL2, RORA, and RORB (60), while CLOCK, ARNTL, and NPAS2 polymorphisms also relate to seasonal appetite and weight variation (27). These overlapping associations suggest that carriers of specific circadian variants may be simultaneously predisposed to photosensitive mood cycling, seasonal weight gain, and metabolic dysregulation—a biologically coherent diathesis. The absence of genome-wide-significant SAD loci to date likely reflects small common-variant effects and gene-environment interactions rather than absence of genetic architecture (59).

A molecular rationale links two of these genes directly to the inflammatory and metabolic core of the SMO hypothesis, making them more than incidental statistical signals. ZBTB20, the strongest signal in the first SAD GWAS (59) and a contributor to sex-specific seasonality risk (58), is a transcriptional repressor with pleiotropic control over precisely the systems SMO implicates: it is required for normal postnatal glucose homeostasis, and in innate-immune cells it represses IκBα transcription and thereby promotes Toll-like-receptor-driven NF-κB activation and pro-inflammatory cytokine output (61). A variant that simultaneously biases glucose handling and amplifies NF-κB-dependent inflammation is a plausible single node coupling seasonal adiposity to the neuroinflammatory cascade central to the model. NPAS2, in turn, is not only the circadian variant most robustly associated with seasonal pattern in BD (60), but also one of three clock genes (with PER2 and ARNTL) experimentally implicated in winter depression (62), and its role in energy regulation and feeding behavior connects the circadian clock to the appetite-weight axis. We therefore propose that ZBTB20 and NPAS2 are candidate pleiotropic substrates through which a single genetic diathesis can drive seasonal mood cycling, inflammatory reactivity, and metabolic oscillation in parallel.

Beyond these associations, we specify how the ZBTB20 and NPAS2 hypotheses can be tested at variant and expression resolution rather than at the candidate-gene level. First, the lead SAD/seasonality variants at ZBTB20 and the seasonal-pattern signals at NPAS2 can be interrogated for cis-expression quantitative trait loci (cis-eQTLs) in disease-relevant tissues—hypothalamus and other brain regions, adipose tissue, and innate-immune cells—using resources such as GTEx and eQTLGen (63, 64) testing whether risk alleles predict the direction of expression change the model implies (for ZBTB20, altered glucose handling together with de-repressed NF-κB–driven inflammation; for NPAS2, altered clock and energy-regulatory output). Second, statistical colocalization (e.g., coloc or summary-based Mendelian randomization) (65, 66) can test whether the SAD/BD–seasonal-pattern association and the adiposity- or inflammation-related eQTL signals share a single causal variant at each locus, distinguishing genuine pleiotropy from linkage-disequilibrium coincidence. Third, a transcriptome-wide association study (TWAS) (67) can link genetically predicted expression in brain, adipose, and immune tissues to seasonal mood and metabolic phenotypes. Fourth, Mendelian randomization (68) using adiposity and inflammation instruments—independent of season by construction—can test directional liability to SAD and to BD with seasonal pattern, with reverse-direction analyses probing a mood-to-adiposity path. We present these as the population-genetic complement to the within-subject physiological tests of Section 6, and note that current SAD genetic evidence remains largely candidate-gene-level, so these analyses are proposed confirmations rather than established results.

## 5 The seasonal metabolic oscillation hypothesis: integrated model, assumptions, and falsifiability

Before detailing the integrated loop, we make explicit which parts of the model are load-bearing and which are auxiliary, so that the framework’s breadth is not mistaken for a claim that all mechanisms must simultaneously hold. The model has a two-node explanatory core: (i) winter adipose expansion driving low-grade neuroinflammation and IDO1 activation with a kynurenine-pathway shift, and (ii) leptin resistance and HPA-axis dysregulation acting on the same depressive node. Everything else—serotonergic self-medication, the exploratory dopaminergic/summer arm, the pars tuberalis–tanycyte thyroid switch as an upstream photoperiodic driver, and the circadian/reward genetic diathesis—is treated as context, upstream input, moderator, or exploratory extension rather than as a co-equal mechanism. The falsifiable core specified in Section 5.1 concerns only this two-node pathway: disconfirmation of an auxiliary arm would trim the model without refuting it, whereas failure of the core would falsify SMO specifically.

Integrating the foregoing evidence, we propose the SMO hypothesis (**Figure 1**). In individuals carrying circadian-clock and/or reward-system variants, seasonal changes in light availability drive not only circadian misalignment but also a reproducible metabolic cycle. In winter, reduced photostimulation impairs entrainment, extends melatonin secretion, reduces satiety signaling, promotes carbohydrate-driven hyperphagia, and expands adipose tissue; this adipose burden activates low-grade neuroinflammation and IDO1 (lowering serotonin and raising neurotoxic kynurenines) together with leptin resistance and HPA hyperactivity—consolidating the winter depressive episode. We deliberately omit any winter D2-receptor change from this depressive node: direct seasonal imaging indicates that striatal D2/3 availability is, if anything, higher under short winter photoperiods (50), so dopaminergic receptor dynamics are treated as a photoperiod-driven, autoreceptor-mediated process running in parallel to the adiposity loop rather than as part of it. In spring-summer, lengthening photoperiod restores entrainment and suppresses melatonin while negative energy balance reduces adiposity, reversing the inflammatory-metabolic burden, restoring serotonergic tone, and—through photoperiodic effects on dopamine synthesis and turnover rather than adiposity-driven receptor up-regulation—lowering the threshold for hypomanic elevation in predisposed individuals.

Existing frameworks linking metabolic syndrome to mood disorders emphasize chronic low-grade inflammation and oxidative stress (69) and insulin resistance as mediators of depression risk (70). The SMO hypothesis extends these by adding a seasonal temporal dimension: it is not chronic metabolic dysfunction per se but its cyclical oscillation—amplified by photoperiodic drivers—that is proposed to generate the characteristic mood cycling of SAD and of BD with SP. We emphasize that SMO is not advanced as a rival to the circadian, serotonergic, and photic models but as a metabolic layer that operates alongside and downstream of them: photoperiod remains the shared upstream driver, and the established circadian and light-based mechanisms are best regarded as co-acting, higher-order explanations rather than competitors. **Table 1** compares the explanatory scope of existing models with that of SMO in this complementary spirit. We stress a corresponding evidentiary caveat: most of the mechanistic data marshaled above derive from chronic obesity and high-fat-diet models, so the claim that the same pathways operate within a months-long seasonal amplitude is an extrapolation; the seasonal-oscillation-specific evidence (21, 27) is comparatively sparse and constitutes the most important target for confirmation.

**Table 1:** Explanatory scope of existing models of seasonal affective disorder compared with the seasonal metabolic oscillation (SMO) hypothesis.

Model	Core mechanism	Winter depression?	Summer switch?	Weight cycling?/key limitation
Circadian phase-shift	Melatonin/pacemaker desynchronization	Yes	Partly	No/complementary upstream account; does not address the metabolic oscillation, which SMO adds
Serotonergic (SERT)	Elevated winter SERT, lower synaptic 5-HT	Yes	Weak	No/compatible with SMO as one node; tryptophan depletion alone did not reproduce relapse
Light/photon deficiency	Reduced photic input	Yes	Partly	No/shared photic driver; SMO extends it to the appetite-weight axis
Metabolic-syndrome inflammation (chronic)	Chronic low-grade inflammation/insulin resistance	Partly	No	Static/shares SMO’s mechanisms but lacks the seasonal temporal dimension SMO adds
SMO (proposed)	Seasonal adiposity oscillation → neuroimmune, neuroendocrine, dopaminergic cycle	Yes	Yes	Yes/central causality not yet tested

The SMO hypothesis also sits naturally within an evolutionary framework that has recently been articulated for mood disorders. Under the “metabolic plasticity” account, the seasonal coupling of energy metabolism, circadian timing, and behavior is not a malfunction but a conserved adaptation: ancestral organisms met predictable seasonal scarcity by shifting fuel utilization, accumulating adipose reserves in autumn-winter, and entering states of reduced activity and energy conservation, with these programs entrained by photoperiod (71). On this view, the winter SAD phenotype—hyperphagia, carbohydrate craving, weight gain, hypersomnia, and psychomotor slowing—recapitulates a hibernation-like energy-conservation strategy that becomes maladaptive in the modern environment of caloric abundance, artificial light, and year-round demand. The SMO hypothesis specifies the proximate machinery through which such an ancestral seasonal energy program could express itself as mood pathology: the same adiposity oscillation that once buffered winter scarcity now drives a neuroinflammatory and neuroendocrine cascade in genetically susceptible individuals.

The principal alternative to the SMO model is that photoperiod is a common cause of both adiposity change and mood change rather than adiposity being a mediator between them. Lengthening or shortening daylight could independently entrain mood (an established effect) and energy balance, producing tightly correlated seasonal time-courses with no causal arrow from fat to brain. This common-driver account is difficult to exclude with observational data alone, because both variables are entrained to the same exogenous oscillator; even cross-lagged longitudinal designs can yield spurious temporal precedence under shared seasonal forcing. Distinguishing mediation from common causation therefore requires experimentally decoupling adiposity from photoperiod—for example, manipulating energy balance while holding season (and measured light exposure) constant, or studying populations at low latitude or under controlled lighting. We treat the experimental-medicine design (Section 6) as the primary causal test for this reason.

The model is consistent with longitudinal epidemiology. A 31-year Finnish registry study of 978,079 hospitalizations found that manic admissions peak in summer and reach a nadir in winter, while bipolar depressive and mixed admissions peak in autumn and spring respectively (72). The COBY study showed that depressive episodes are more frequent in winter and hypomanic/manic episodes in summer in youth with bipolar spectrum disorder, with latitude moderating the effect, and the Prechter Longitudinal Study found that higher seasonality scores were associated with greater mood-state variance (73).

### 5.1 Assumptions and boundary conditions

The hypothesis rests on four explicit assumptions, each of which is empirically addressable: (1) Temporal precedence: seasonal weight change is causally upstream of mood change rather than merely concurrent or consequent. (2) Sufficiency: even subclinical seasonal increases in adiposity generate enough adipose inflammation to shift central neurochemistry. (3) Co-aggregation: the same individuals carry both the circadian/reward diathesis and the metabolically reactive phenotype. (4) Reversibility magnitude: the degree of summer weight loss is sufficient to meaningfully reverse adiposity-driven IDO1 activation, the kynurenine-pathway shift, and leptin/HPA dysregulation.

Assumptions 2 and 4 are quantitative and currently unproven: the typical seasonal weight swing in SAD is modest (on the order of a few kilograms), and it is not yet established that an adiposity change of this magnitude is sufficient to move central inflammatory, kynurenine, or dopaminergic indices. The inverse relationship between the winter-to-summer weight differential and plasma adiponectin reported even at subclinical levels (21) is a preliminary indication that small seasonal swings carry measurable adipose-endocrine signal, but a formal dose–response relationship between seasonal adiposity amplitude and central neurochemistry remains to be demonstrated and should be treated as a hypothesis rather than a premise.

We wish to be unambiguous that this sufficiency–reversibility question is the single most important unproven premise of the entire model, not a peripheral caveat: if a seasonal fat-mass swing of the magnitude actually observed in SAD—on the order of only a few kilograms—cannot move central inflammatory, kynurenine, leptin, and HPA indices by a meaningful amount, then the mediating role of adiposity fails regardless of how well the remaining associations hold. We therefore commit in Section 6 to prespecified target magnitudes for both the adiposity swing and the downstream biomarker changes, so that this assumption is stated as a falsifiable quantitative prediction rather than assumed.

Because the model invokes several parallel mechanisms, it risks being over-determined—disconfirmation of one arm would leave the others intact. We therefore specify a minimal falsifiable core that is unique to SMO and distinct from a generic inflammation-depression account: within the same individuals, the seasonal change in adiposity must temporally precede, and statistically account for, a meaningful share of the seasonal change in mood polarity. To make this operational rather than rhetorical, we propose prespecified criteria for what would count as support: (i) directionality and lag—change in adiposity (DXA-measured fat mass) should lead change in mood ratings by a defined interval on the order of two to six weeks in cross-lagged models, not merely co-vary concurrently; (ii) effect size—after covarying measured light exposure and physical activity, seasonal adiposity change should mediate a prespecified minimum proportion (we suggest ≥20%) of the variance in seasonal mood change, with the indirect path significant; (iii) biomarker coherence—the mediated path should be accompanied by seasonal oscillation, in the predicted directions, of at least the inflammatory (hsCRP, IL-6, TNF-α) and kynurenine/tryptophan indices; and (iv) comparison groups—the coupling should be stronger in hyperphagic winter-pattern SAD and seasonal-pattern Bipolar II than in non-seasonal mood-disorder and healthy controls. Effects below these thresholds, or abolition of mediation once photoperiod and activity are modeled, would weaken or falsify the core. Failure of the minimal core—no temporal precedence and no mediation—would falsify SMO specifically, not merely one of its arms.

## 6 Testable predictions and study designs

The central prediction is that, within the same individuals, seasonal swings in adiposity, inflammatory and kynurenine markers, and dopaminergic indices track the timing and polarity of mood episodes. Several complementary designs, each with explicit confirmation and falsification criteria, would test it (summarized in **Table 2**).

**Table 2:** Proposed empirical designs for testing the SMO hypothesis, with explicit confirmation and falsification criteria.

Design	Population	Key measured variables	Confirmation criterion	Falsification criterion
Experimental-medicine weight-stabilization trial	BD/SAD with seasonal weight cycling; first-episode or drug-naïve prioritized	Adiposity (BMI, waist, DXA/VAT, leptin), kynurenine/tryptophan ratio, inflammatory markers, mood ratings across a photoperiodic transition	Preventing seasonal adiposity gain attenuates the expected seasonal shift in inflammatory and mood indices	Mood/inflammatory cycling persists despite stabilized adiposity
Within-subject longitudinal cohort	Seasonally cycling BD-II/SAD followed across ≥1 full year	Repeated adiposity, IDO1/kynurenine metabolites, leptin, mood load, plus light dosimetry, temperature, melatonin timing, harmonic/cosinor covariates	Within-person adiposity increase precedes and predicts depressive worsening; spring loss precedes hypomanic features in carriers	No temporal coupling between adiposity change and subsequent mood change
Molecular neuroimaging (PET)	Within-subject winter vs summer states	Adiposity, hypomanic symptoms, TSPO-PET neuroinflammation, [18F]FDOPA synthesis and DAT as primary dopaminergic endpoints; [11C]raclopride D2/3 as secondary	Seasonal change in dopamine synthesis/DAT and TSPO signal tracks adiposity and mood change	No seasonal change in dopaminergic indices disconfirms the arm
Population genetics (MR/coloc/TWAS)	Population-scale genetic cohorts	Shared circadian and adiposity variants (incl. ZBTB20, NPAS2); cis-eQTLs, colocalization, TWAS in brain/adipose/immune tissue	Adiposity/circadian variants causally predict seasonal mood phenotypes	No causal effect of adiposity/circadian variants on seasonal mood at the population level

To make the sufficiency assumption quantitative and powerable, we prespecify working targets. We hypothesize that a winter-to-summer swing on the order of 2–4 kg total fat mass, or approximately 0.5–1.5 kg (roughly 10–20%) of visceral adipose tissue (VAT) measured by DXA or MRI, sustained across the 8–16-week photoperiodic transition, represents the minimum amplitude capable of producing detectable central-relevant change (20, 21). Anchored to this, the predicted seasonal biomarker shifts are approximately 0.4–0.6 mg/L in hsCRP, 15–25% in IL-6 and TNF-α, and ≥15% in the kynurenine/tryptophan ratio, together with fat-mass-scaled changes in leptin and measurable change in cortisol dynamic-feedback indices. Because these designs are within-subject, they are efficiently powered: detecting a within-person correlation of r ≈ 0.4–0.5 between seasonal ΔVAT and seasonal Δ(kynurenine/tryptophan) at α = 0.05 with 80–90% power requires roughly 30–50 participants, while the mediation target (seasonal adiposity change accounting for ≥20% of seasonal mood variance with a significant indirect path) is powered at approximately 50–80 participants depending on the mediator–outcome association. These magnitudes are hypothesized targets extrapolated from chronic-obesity effect sizes (36) and are themselves falsifiable: seasonal fat-mass swings that fail to move these biomarkers by the stated amounts would weaken the sufficiency assumption and, with it, the core model.

### 6.1 Experimental-medicine trial (primary causal test)

In individuals with SAD, a randomized trial of a weight-stabilizing or metabolic intervention (structured caloric/macronutrient control, metformin, or a GLP-1 receptor agonist) applied across winter, with mood-cycling and biomarker outcomes, tests whether attenuating the metabolic oscillation reduces cycling. Crucially, this design experimentally decouples adiposity from photoperiod—altering energy balance while season and measured light exposure are held constant—and is therefore the only proposed test that can distinguish mediation from photoperiodic common causation. A complementary low-latitude or controlled-lighting arm would further separate the two. The model predicts that flattening the adiposity oscillation attenuates mood cycling; no effect on cycling despite successful weight stabilization would weaken the core claim.

To operationalize and verify weight stabilization without introducing confounds, stabilization is defined *a priori* as maintenance of body weight within ±1 kg and fat mass within a prespecified DXA band across the winter window, verified by repeated DXA (with bioimpedance between scans) and frequent weigh-ins. Dietary composition and physical activity—each an independent influence on inflammation and mood—are held constant and measured rather than left free: isocaloric-target counseling with fixed macronutrient proportions, weighed or application-based dietary records, and continuous actigraphy with light dosimetry, so that diet and activity enter the analysis as covariates. Because the behavioral/caloric, metformin, and GLP-1-receptor-agonist arms carry different confound profiles, they are run as separate arms so that any mood effect is attributable to the metabolic manipulation rather than to a specific agent. Given that the model itself predicts that rapid, photoperiod-coupled weight loss could lower the threshold for hypomanic elevation in predisposed individuals, and that GLP-1-associated manic emergence has been reported (55), safety provisions for bipolar participants are treated as a core design feature: enrolment on stable mood-stabilizing treatment where clinically indicated, frequent prospective mood monitoring (clinician-rated YMRS and MADRS plus daily self-ratings, with actigraphy as an objective marker of emerging (hypo)mania), prespecified stopping and de-escalation rules, avoidance of rapid weight-loss trajectories in favor of controlled stabilization, and independent data-safety monitoring.

### 6.2 Within-subject longitudinal cohort

Individuals with SAD are assessed repeatedly across seasons (winter depressive vs. summer remission) with body-fat measurement (DXA), an inflammatory panel (IL-6, TNF-α, hsCRP), the kynurenine/tryptophan ratio, leptin, mood ratings, and objective light exposure and actigraphy. Cross-lagged panel or mediation analysis tests temporal precedence—the model’s minimal core. The model predicts directional, lagged coupling from adiposity to subsequent mood change; absence of such precedence would falsify the minimal core.

Because adiposity and mood are both entrained to daylength, temporal precedence in cross-lagged models is necessary but not sufficient for mediation. To separate an adiposity-mediated path from parallel photoperiodic forcing, the cohort incorporates direct personal light exposure (wrist or pendant dosimetry) and ambient temperature as measured, time-varying covariates rather than season inferred from the calendar, alongside actigraphy and salivary dim-light melatonin onset. Analyses include harmonic/cosinor terms for photoperiod and measured light and test whether the adiposity→mood indirect path survives their inclusion; the informative signal is the portion of the adiposity time-course that deviates from the pure photoperiodic harmonic. Two quasi-experimental levers further help decouple the two: low-latitude or controlled-lighting cohorts, in which photoperiod amplitude is compressed, and the genetic-instrument analyses below, in which adiposity-raising variants are independent of season. Convergence across the decoupling trial, the light-covaried longitudinal mediation, and the genetic instruments is what would license a causal reading; divergence would not.

### 6.3 Molecular neuroimaging

Consistent with the revised dopaminergic account (Section 4.4), the imaging arm prioritizes presynaptic synthesis and transporter measures over receptor availability, because direct seasonal PET shows higher—not lower—striatal D2/3 binding under short winter photoperiods and the model therefore does not predict an adiposity-driven summer receptor rebound (50). The primary dopaminergic endpoint is dopamine synthesis capacity by [^18^F]-FDOPA PET; dopamine-transporter binding ([¹²³I]-FP-CIT SPECT or [¹¹C]-PE2I PET) is the transporter measure; and [¹¹C]-raclopride D2/3 availability is retained only as a secondary contrast endpoint. TSPO-PET indexes neuroinflammation in the same individuals, linking the dopaminergic and inflammatory arms. To test lagged rather than merely state-dependent relationships, paired winter-depressive and summer-remission scans are embedded within the longitudinal cohort, so that DXA fat mass, inflammatory and kynurenine panels, leptin, actigraphy, personal light dosimetry, and salivary melatonin are sampled densely (every 2–4 weeks) around each scan, permitting change-on-change modeling with defined 2–6-week lags between the adiposity, light, and melatonin time-courses and the dopaminergic endpoints. Absence of any seasonal change in dopamine synthesis, DAT, or parallel neuroinflammation would disconfirm this arm.

Prespecified subgroups. We further prespecify directional hypotheses for subgroups, because differential dynamics are themselves tests of the model. Given the female preponderance of SAD (6) and the sex-specific ZBTB20 effect (58), we expect stronger adiposity–mood coupling in women and enter sex as a moderator. Greater baseline adiposity and insulin resistance are hypothesized to amplify the oscillation (20), so participants are stratified by baseline BMI and HOMA-IR. The model is specified for the hyperphagic/atypical winter phenotype and predicts weak or absent coupling in melancholic, non-hyperphagic presentations, which serve as an internal negative control. Because seasonal pattern is most strongly associated with Bipolar II (11), we predict the clearest adiposity-linked cycling in Bipolar II and seasonal-pattern MDD, with Bipolar I as a contrast. These stratifications shape the design by requiring adequate per-stratum sampling (enriching for hyperphagic winter-pattern SAD and Bipolar II), prespecified moderator analyses (sex, latitude, baseline BMI, and DRD4-7R/ZBTB20/NPAS2 genotype), and the use of non-hyperphagic and summer-pattern (reverse-seasonal) SAD as boundary conditions that delimit the model’s scope.

Across all designs, prespecified moderators (sex, latitude, baseline BMI, and DRD4-7R, ZBTB20, and NPAS2 genotype) and mediators (kynurenine/tryptophan ratio, leptin signaling, dopaminergic indices) should be tested explicitly. In addition, Mendelian randomization using shared circadian and adiposity variants could help adjudicate causal direction at the population level, and genetic studies could test whether ZBTB20 and NPAS2 variants are co-enriched in individuals with both seasonal mood cycling and metabolic phenotypes. As detailed in Section 4.5, these population-genetic tests should proceed at variant and expression resolution—cis-eQTL interrogation, statistical colocalization, TWAS, and bidirectional Mendelian randomization—rather than at the candidate-gene level, so that shared genetic liability to adiposity/inflammation and to seasonal mood phenotypes can be distinguished from linkage-disequilibrium coincidence. Across all designs, sampling first-episode or drug-naïve participants is a methodological priority rather than a convenience: weight-inducing atypical antipsychotics and several mood stabilizers themselves expand adiposity and perturb glucose and inflammatory indices, and their inclusion would confound the very adiposity-to-mood path the model seeks to isolate. Where fully drug-naïve cohorts are not feasible, current medication and its metabolic liability must be explicitly modeled as covariates.

## 7 Clinical and translational implications

If the SMO hypothesis is supported, several implications follow, stated here conditionally in proportion to current evidence. First, individuals with SAD may warrant metabolic screening—BMI, waist circumference, fasting glucose, HbA1c, and lipids—during seasonal depressive episodes, consistent with broader recommendations for metabolic monitoring in mood disorders (74, 75); seasonal fluctuations in these indices might serve as both risk markers and therapeutic targets.

Second, pharmacotherapy could be weight-conscious in this population. Bupropion has demonstrated efficacy in SAD prophylaxis while typically producing weight loss rather than gain (76, 77), whereas agents with substantial weight-gain liability—valproate, olanzapine, quetiapine—could, in principle, exacerbate the adipose-driven mechanisms proposed here (74, 78–80); lithium and lamotrigine show more favorable weight profiles (78–80). Third, bariatric surgery reduces depressive-symptom burden alongside weight loss (SMD ≈ −0.87 across 33 studies) with effects maintained long-term (81, 82), and metformin and GLP-1 receptor agonists have been proposed as adjuncts in BD with metabolic comorbidity (78); their potential to attenuate seasonal metabolic amplification of mood cycling warrants investigation, with the caveat noted above regarding manic emergence. The bariatric finding raises an apparent tension with the model: if weight loss lowers the threshold for mood elevation, why does surgical weight loss generally improve rather than destabilize mood? The SMO framework would attribute the difference to context rather than to weight loss per se—surgical loss occurs once, gradually, outside a photoperiodic transition, and predominantly in individuals without a bipolar-spectrum diathesis, whereas the model’s hypomanic arm is specified for predisposed carriers undergoing a rapid spring photoperiod-coupled reversal. This account is *post hoc* and itself testable: it predicts that mood elevation after weight loss should concentrate in diathesis carriers and in spring-timed loss, a prediction the designs in Section 6 can address.

Fourth, chronotherapeutic strategies—bright-light therapy, melatonin, and sleep-phase advancement—may target not only the circadian arm but, indirectly, the metabolic arm. Melatonin administration in circadian disruption reduced leptin and insulin, suggesting that circadian realignment exerts metabolic effects (83), and adipose circadian dysfunction has been proposed as a bridge between circadian disruption and metabolic disease, with chronotherapy potentially improving glucose control, lipids, and weight (84, 85). Combining light therapy with structured dietary timing and metabolic monitoring may therefore be more comprehensive than photic intervention alone.

## 8 Limitations and evidence quality

Several limitations and alternative explanations must be acknowledged. First, the causal role of weight change in mood cycling has not been directly tested; weight gain can follow manic episodes (86), so reverse causation—mood states driving eating and weight—remains a live alternative. Second, seasonal oscillation of kynurenine-pathway activity and dopaminergic availability has not been characterized in SAD. Third, some individuals show pronounced seasonal mood cycling without substantial weight variation, indicating that adiposity is likely a moderating rather than universal variable. Fourth, rapid tryptophan depletion did not reproduce depressive relapse in SAD (87), arguing against serotonin as the sole node and supporting the multi-pathway structure proposed here. Fifth, genetic evidence for shared circadian architecture remains largely at the candidate-gene level (59). Sixth, and most fundamentally, photoperiod is a plausible common cause of both adiposity and mood change, so observed seasonal co-variation need not reflect a causal path from fat to brain; only experimentally decoupling the two (Section 6) can adjudicate this. Seventh, the direct evidence for the hypomanic arm comes from small hypothesis-consistent case reports (54, 55), which are particularly vulnerable to publication and positive-result bias and carry their own confounds. Eighth, summer-pattern (reverse-seasonal) SAD and non-hyperphagic SAD sit uneasily with a uniformly adiposity-driven account and are better treated as boundary conditions than as supporting cases. Finally, most of the supporting literature derives from high-latitude, predominantly Western populations, limiting generalizability across latitude, ancestry, and cultural context.

Evidence quality is uneven across the model. The phenomenological and bidirectional-epidemiology claims rest on meta-analyses and large cohorts, whereas the dopaminergic arm rests on small, uncontrolled observations and extrapolated imaging. Much of the seasonality literature relies on retrospective self-report instruments (e.g., the Seasonal Pattern Assessment Questionnaire), which are subject to recall and expectancy bias; diagnostic heterogeneity, variable BMI/adiposity measures, medication confounding, and the limited translatability of high-fat-diet rodent models further temper inference. These caveats define where stronger, prospective, within-subject data are most needed.

## 9 Conclusion

In conclusion, SAD has long been conceptualized primarily as a disorder of light and circadian timing. We propose that seasonal adiposity is an underrecognized neurobiological contributor to—and candidate modulator of—the expression, severity, and polarity of seasonal mood cycling, acting through neuroinflammation-driven kynurenine activation, leptin resistance and HPA dysregulation, and—most tentatively—obesity-associated dopaminergic change, all reversing with the spontaneous adiposity loss of summer, on a substrate of shared circadian genetic vulnerability. The strength of these arms is uneven: the winter-depression metabolic-amplification arm is the better supported, whereas the summer-hypomania dopaminergic arm remains exploratory and awaits direct test. We reiterate that the causal architecture of the model remains provisional: the temporal-precedence and sufficiency assumptions are experimentally undemonstrated, photoperiod is a plausible common cause of both adiposity and mood, and the experimental-medicine trial of Section 6—which holds season and measured light exposure constant while manipulating energy balance—is the decisive test that could confirm or refute the mediating role of adiposity. If validated, the SMO hypothesis may reframe SAD as a recurrent metabolic-neuroimmune-mood disorder and open therapeutic avenues that integrate metabolic medicine with established chronobiological and psychopharmacological approaches.

## Data Availability

The original contributions presented in the study are included in the article/supplementary material. Further inquiries can be directed to the corresponding author.

## References

[1] Nevarez Flores AG Bostock EC Neil AL . Should clinicians and the general population be concerned about seasonal affective disorder in Australia? Med J Aust. (2022) 216:507–9. doi: 10.5694/mja2.51518 35524436

[2] Dollish HK Tsyglakova M McClung CA . Circadian rhythms and mood disorders: Time to see the light. Neuron. (2024) 112:25–40. doi: 10.1016/j.neuron.2023.09.023 37858331 PMC10842077

[3] Rohan KJ Terman JM Iyiewuare P Perez J Camuso JA Postolache TT . Prospectively assessed summer mood status in major depression, recurrent with seasonal pattern: Evidence for SAD’s construct validity. J Affect Disord. (2024) 349:32–8. doi: 10.1016/j.jad.2023.12.070 38160889 PMC10923172

[4] Field T . Seasonal affective disorder: A narrative review. J Clin Psychol Neurol. (2024) 1. doi: 10.61440/JCPN.2024.v2.14

[5] Kim K Kim J Jung S Kim H-W Kim H-S Son E . Global prevalence of seasonal affective disorder by latitude: A systematic review and meta-analysis. J Affect Disord. (2025) 390:119807. doi: 10.1016/j.jad.2025.119807 40614973

[6] Yasmin H . Epidemiological study on the prevalence of winter depression in Pakistan. J Dev Soc Sci. (2022) 3. doi: 10.47205/jdss.2022(3-II)59 41876239

[7] Jahan-Mihan A Stevens P Medero-Alfonso S Brace G Overby LK Berg K . The role of water-soluble vitamins and vitamin D in prevention and treatment of depression and seasonal affective disorder in adults. Nutrients. (2024) 16:1902. doi: 10.3390/nu16121902 38931257 PMC11206829

[8] Nevarez-Flores AG Bostock ECS Neil AL . The underexplored presence of seasonal affective disorder in the southern hemisphere: A narrative review of the Australian literature. J Psychiatr Res. (2023) 162:170–9. doi: 10.1016/j.jpsychires.2023.05.003 37167837

[9] Yeom JW Lee J Park S Cho C Lee H . Optimizing winter‐type seasonality criteria in East Asian populations: A machine learning approach using the seasonal pattern assessment questionnaire. Brain Behav. (2025) 15:e70834. doi: 10.1002/brb3.70834 40898717 PMC12405589

[10] Zelinski L Pálmadóttir AD Birgisdóttir ÁG Sesseljuson LD Ólafsson RP Bathke AC . Prevalence of seasonal affective disorder and seasonality of mood and behaviour in Iceland – Methodological implications for revisiting the latitude hypothesis. J Affect Disord. (2026) 398:121057. doi: 10.1016/j.jad.2025.121057 41461237

[11] Yeom JW Cho C Jeon S Seo JY Son S Ahn Y . Bipolar II disorder has the highest prevalence of seasonal affective disorder in early‐onset mood disorders: Results from a prospective observational cohort study. Depress Anxiety. (2021) 38:661–70. doi: 10.1002/da.23153 33818866

[12] Fico G de Toffol M Anmella G Sagué‐Vilavella M Dellink A Verdolini N . Clinical correlates of seasonality in bipolar disorder: A specifier that needs specification? Acta Psychiatr Scand. (2021) 143:162–71. doi: 10.1111/acps.13251 33140436

[13] Bostan S Melhuish Beaupre LM Ercis M Coombes BJ Romo‐Nava F Prieto ML . Seasonality patterns in bipolar disorder: Associations with clinical phenotypes and treatment‐related outcomes. Acta Psychiatr Scand. (2026) 153:257–69. doi: 10.1111/acps.70063 41450248

[14] Geoffroy PA Bellivier F Scott J Etain B . Seasonality and bipolar disorder: A systematic review, from admission rates to seasonality of symptoms. J Affect Disord. (2014) 168:210–23. doi: 10.1016/j.jad.2014.07.002 25063960

[15] Lewy AJ Lefler BJ Emens JS Bauer VK . The circadian basis of winter depression. Proc Natl Acad Sci. (2006) 103:7414–9. doi: 10.1073/pnas.0602425103 16648247 PMC1450113

[16] Willeit M Praschak-Rieder N Neumeister A Zill P . A polymorphism (5-HTTLPR) in the serotonin transporter promoter gene is associated with DSM-IV depression subtypes in seasonal affective disorder. Mol Psychiatry. (2003) 8:942–6. doi: 10.1038/sj.mp.4001392 14593433

[17] Golden RN Gaynes BN Ekstrom RD Hamer RM Jacobsen FM Suppes T . The efficacy of light therapy in the treatment of mood disorders: A review and meta-analysis of the evidence. Am J Psychiatry. (2005) 162:656–72. doi: 10.1176/appi.ajp.162.4.656 15800134

[18] Rosenthal NE . Seasonal affective disorder. Arch Gen Psychiatry. (1984) 41:72. doi: 10.1001/archpsyc.1984.01790120076010 6581756

[19] Yang Y Zhang S Zhang X Xu Y Cheng J Yang X . The role of diet, eating behavior, and nutrition intervention in seasonal affective disorder: A systematic review. Front Psychol. (2020) 11. doi: 10.3389/fpsyg.2020.01451 32903693 PMC7438823

[20] Kräuchi K Reich S Wirz-Justice A . Eating style in seasonal affective disorder: Who will gain weight in winter? Compr Psychiatry. (1997) 38:80–7. doi: 10.1016/S0010-440X(97)90085-7 9056125

[21] Akram F Gragnoli C Raheja UK Snitker S Lowry CA Stearns-Yoder KA . Seasonal affective disorder and seasonal changes in weight and sleep duration are inversely associated with plasma adiponectin levels. J Psychiatr Res. (2020) 122:97–104. doi: 10.1016/j.jpsychires.2019.12.016 31981963 PMC7024547

[22] Regmi P Young M Minigo G Milic N Gyawali P . Photoperiod and metabolic health: Evidence, mechanism, and implications. Metabolism. (2024) 152:155770. doi: 10.1016/j.metabol.2023.155770 38160935

[23] Reutrakul S Van Cauter E . Sleep influences on obesity, insulin resistance, and risk of type 2 diabetes. Metabolism. (2018) 84:56–66. doi: 10.1016/j.metabol.2018.02.010 29510179

[24] Wurtman RJ Wurtman JJ . Carbohydrate craving, obesity and brain serotonin. Appetite. (1986) 7:99–103. doi: 10.1016/S0195-6663(86)80055-1 3527063

[25] Levitan RD Masellis M Lam RW Kaplan AS Davis C Tharmalingam S . A birth-season/DRD4 gene interaction predicts weight gain and obesity in women with seasonal affective disorder: A seasonal thrifty phenotype hypothesis. Neuropsychopharmacology. (2006) 31:2498–503. doi: 10.1038/sj.npp.1301121 16760922

[26] Levitan RD Masellis M Basile VS Lam RW Kaplan AS Davis C . The dopamine-4 receptor gene associated with binge eating and weight gain in women with seasonal affective disorder: An evolutionary perspective. Biol Psychiatry. (2004) 56:665–9. doi: 10.1016/j.biopsych.2004.08.013 15522250

[27] Kim H-I Lee H-J Cho C-H Kang S-G Yoon H-K Park Y-M . Association of CLOCK, ARNTL , and NPAS2 gene polymorphisms and seasonal variations in mood and behavior. Chronobiol Int. (2015) 32:785–91. doi: 10.3109/07420528.2015.1049613 26134245

[28] Luppino FS de Wit LM Bouvy PF Stijnen T Cuijpers P Penninx BWJH . Overweight, obesity, and depression. Arch Gen Psychiatry. (2010) 67:220. doi: 10.1001/archgenpsychiatry.2010.2 20194822

[29] Mannan M Mamun A Doi S Clavarino A . Is there a bi-directional relationship between depression and obesity among adult men and women? Systematic review and bias-adjusted meta analysis. Asian J Psychiatr. (2016) 21:51–66. doi: 10.1016/j.ajp.2015.12.008 27208458

[30] Pan A Sun Q Czernichow S Kivimaki M Okereke OI Lucas M . Bidirectional association between depression and obesity in middle-aged and older women. Int J Obes. (2012) 36:595–602. doi: 10.1038/ijo.2011.111 21654630 PMC3233636

[31] Calkin C Van De Velde C Růžičková M Slaney C Garnham J Hajek T . Can body mass index help predict outcome in patients with bipolar disorder? Bipolar Disord. (2009) 11:650–6. doi: 10.1111/j.1399-5618.2009.00730.x 19689507 PMC3544930

[32] Doane MJ Thompson J Jauregui A Gasper S Csoboth C . Clinical, economic, and humanistic outcomes associated with obesity among people with bipolar I disorder in the United States: Analysis of national health and wellness survey data. ClinicoEconomics Outcomes Res. (2023) 15:681–9. doi: 10.2147/CEOR.S411928 37743958 PMC10516196

[33] Goldstein BI Blanco C He J-P Merikangas K . Correlates of overweight and obesity among adolescents with bipolar disorder in the National Comorbidity Survey–Adolescent Supplement (NCS-A). J Am Acad Child Adolesc Psychiatry. (2016) 55:1020–6. doi: 10.1016/j.jaac.2016.08.010 27871636

[34] Jerrell JM McIntyre RS Tripathi A . A cohort study of the prevalence and impact of comorbid medical conditions in pediatric bipolar disorder. J Clin Psychiatry. (2010) 71:1518–25. doi: 10.4088/JCP.09m05585ora 20584522

[35] Bartoli F Cavaleri D Callovini T Palpella D Piacenti S Crocamo C . Clinical and metabolic correlates of DSM-5 mixed features in subjects with bipolar depression and mania: A cross-sectional study. J Psychosom Res. (2025) 188:111990. doi: 10.1016/j.jpsychores.2024.111990 39591804

[36] de A. Boleti A de O. Cardoso P Frihling BF e Silva P de Moraes LRN Migliolo L . Adipose tissue, systematic inflammation, and neurodegenerative diseases. Neural Regener Res. (2023) 18:38. doi: 10.4103/1673-5374.343891 35799506 PMC9241402

[37] Almulla AF Thipakorn Y Vasupanrajit A Tunvirachaisakul C Oxenkrug G Al-Hakeim HK . The tryptophan catabolite or kynurenine pathway in a major depressive episode with melancholia, psychotic features and suicidal behaviors: A systematic review and meta-analysis. Cells. (2022) 11:3112. doi: 10.3390/cells11193112 36231075 PMC9563030

[38] Correia AS Vale N . Tryptophan metabolism in depression: A narrative review with a focus on serotonin and kynurenine pathways. Int J Mol Sci. (2022) 23:8493. doi: 10.3390/ijms23158493 35955633 PMC9369076

[39] Castanon N Vancassel S Amadieu C Cussotto S Leyrolle Q Lucas C . Obesity-induced emotional alterations in mice are associated with impairments of tryptophan metabolism along the kynurenine and indole pathways. Brain Behav Immun. (2025) 130:106107. doi: 10.1016/j.bbi.2025.106107 40962034

[40] Maes M . Seasonal variation in plasma L-tryptophan availability in healthy volunteers. Arch Gen Psychiatry. (1995) 52:937. doi: 10.1001/archpsyc.1995.03950230051008 7487342

[41] Hanon EA Lincoln GA Fustin J-M Dardente H Masson-Pévet M Morgan PJ . Ancestral TSH mechanism signals summer in a photoperiodic mammal. Curr Biol. (2008) 18:1147–52. doi: 10.1016/j.cub.2008.06.076 18674911

[42] Ono H Hoshino Y Yasuo S Watanabe M Nakane Y Murai A . Involvement of thyrotropin in photoperiodic signal transduction in mice. Proc Natl Acad Sci. (2008) 105:18238–42. doi: 10.1073/pnas.0808952105 19015516 PMC2587639

[43] Dardente H Hazlerigg DG Ebling FJP . Thyroid hormone and seasonal rhythmicity. Front Endocrinol (Lausanne). (2014) 5. doi: 10.3389/fendo.2014.00019 24616714 PMC3935485

[44] de Assis GG Murawska-Ciałowicz E . Leptin—A potential bridge between fat metabolism and the brain’s vulnerability to neuropsychiatric disorders: A systematic review. J Clin Med. (2021) 10:5714. doi: 10.3390/jcm10235714 34884416 PMC8658385

[45] Milaneschi Y Lamers F Bot M Drent ML Penninx BWJH . Leptin dysregulation is specifically associated with major depression with atypical features: Evidence for a mechanism connecting obesity and depression. Biol Psychiatry. (2017) 81:807–14. doi: 10.1016/j.biopsych.2015.10.023 26742925

[46] Shin AC MohanKumar SMJ Balasubramanian P Sirivelu MP Linning K Woolcock A . Responsiveness of hypothalamo-pituitary-adrenal axis to leptin is impaired in diet-induced obese rats. Nutr Diabetes. (2019) 9:10. doi: 10.1038/s41387-019-0076-y 30886140 PMC6423225

[47] Aschbacher K Rodriguez-Fernandez M van Wietmarschen H Tomiyama AJ Jain S Epel E . The hypothalamic–pituitary–adrenal–leptin axis and metabolic health: A systems approach to resilience, robustness and control. Interface Focus. (2014) 4:20140020. doi: 10.1098/rsfs.2014.0020 25285198 PMC4142017

[48] Wang G-J Volkow ND Logan J Pappas NR Wong CT Zhu W . Brain dopamine and obesity. Lancet. (2001) 357:354–7. doi: 10.1016/S0140-6736(00)03643-6 11210998

[49] Leung C Lutfy K . Dopamine D2 receptors and its downstream signaling in compulsive eating. Brain Sci. (2025) 15:923. doi: 10.3390/brainsci15090923 41008283 PMC12468011

[50] Sun L Malén T Tuisku J Kaasinen V Hietala JA Rinne J . Seasonal variation in D2/3 dopamine receptor availability in the human brain. Eur J Nucl Med Mol Imaging. (2024) 51:3284–91. doi: 10.1007/s00259-024-06715-9 38730083 PMC11369044

[51] Hankir MK Ashrafian H Hesse S Horstmann A Fenske WK . Distinctive striatal dopamine signaling after dieting and gastric bypass. Trends Endocrinol Metab. (2015) 26:223–30. doi: 10.1016/j.tem.2015.03.005 25887491

[52] Mure LS . Intrinsically photosensitive retinal ganglion cells of the human retina. Front Neurol. (2021) 12. doi: 10.3389/fneur.2021.636330 33841306 PMC8027232

[53] Schmidt TM Chen S-K Hattar S . Intrinsically photosensitive retinal ganglion cells: many subtypes, diverse functions. Trends Neurosci. (2011) 34:572–80. doi: 10.1016/j.tins.2011.07.001 21816493 PMC3200463

[54] Palmer CM . Emergence of hypomania and mania following initiation of a ketogenic diet: case series. Front Nutr. (2026) 13. doi: 10.3389/fnut.2026.1815787 42027565 PMC13099860

[55] Romay Gonzalez J Hernández Liebo P Sevilla Diez C Anabitarte Bautista O Cayon de la Hoz L Obeso Menendez R . Weight loss, Semaglutide and Manic Episode”: A case report. Eur Psychiatry. (2024) 67:S210–1. doi: 10.1192/j.eurpsy.2024.452

[56] Garbazza C Benedetti F . Genetic factors affecting seasonality, mood, and the circadian clock. Front Endocrinol (Lausanne). (2018) 9. doi: 10.3389/fendo.2018.00481 30190706 PMC6115502

[57] Etain B Milhiet V Bellivier F Leboyer M . Genetics of circadian rhythms and mood spectrum disorders. Eur Neuropsychopharmacol. (2011) 21:S676–82. doi: 10.1016/j.euroneuro.2011.07.007 21835597

[58] Dang T Russel WA Saad T Dhawka L Ay A Ingram KK . Risk for seasonal affective disorder (SAD) linked to circadian clock gene variants. Biol (Basel). (2023) 12:1532. doi: 10.3390/biology12121532 38132358 PMC10741218

[59] Ho KWD Han S Nielsen JV Jancic D Hing B Fiedorowicz J . Genome-wide association study of seasonal affective disorder. Transl Psychiatry. (2018) 8:190. doi: 10.1038/s41398-018-0246-z 30217971 PMC6138666

[60] Geoffroy PA Lajnef M Bellivier F Jamain S Gard S Kahn J-P . Genetic association study of circadian genes with seasonal pattern in bipolar disorders. Sci Rep. (2015) 5:10232. doi: 10.1038/srep10232 25989161 PMC4437291

[61] Liu X Zhang P Bao Y Han Y Wang Y Zhang Q . Zinc finger protein ZBTB20 promotes toll-like receptor-triggered innate immune responses by repressing IκBα gene transcription. Proc Natl Acad Sci. (2013) 110:11097–102. doi: 10.1073/pnas.1301257110 23776228 PMC3704009

[62] Partonen T Treutlein J Alpman A Frank J Johansson C Depner M . Three circadian clock genes Per2, Arntl, and Npas2 contribute to winter depression. Ann Med. (2007) 39:229–38. doi: 10.1080/07853890701278795 17457720

[63] Aguet F Anand S Ardlie KG Gabriel S Getz GA Graubert A . The GTEx Consortium atlas of genetic regulatory effects across human tissues. Sci (1979). (2020) 369:1318–30. doi: 10.1126/science.aaz1776 32913098 PMC7737656

[64] Võsa U Claringbould A Westra H-J Bonder MJ Deelen P Zeng B . Large-scale cis- and trans-eQTL analyses identify thousands of genetic loci and polygenic scores that regulate blood gene expression. Nat Genet. (2021) 53:1300–10. doi: 10.1038/s41588-021-00913-z 34475573 PMC8432599

[65] Giambartolomei C Vukcevic D Schadt EE Franke L Hingorani AD Wallace C . Bayesian test for colocalisation between pairs of genetic association studies using summary statistics. PloS Genet. (2014) 10:e1004383. doi: 10.1371/journal.pgen.1004383 24830394 PMC4022491

[66] Zhu Z Zhang F Hu H Bakshi A Robinson MR Powell JE . Integration of summary data from GWAS and eQTL studies predicts complex trait gene targets. Nat Genet. (2016) 48:481–7. doi: 10.1038/ng.3538 27019110

[67] Gusev A Ko A Shi H Bhatia G Chung W Penninx BWJH . Integrative approaches for large-scale transcriptome-wide association studies. Nat Genet. (2016) 48:245–52. doi: 10.1038/ng.3506 26854917 PMC4767558

[68] Sanderson E Glymour MM Holmes MV Kang H Morrison J Munafò MR . Mendelian randomization. Nat Rev Methods Primers. (2022) 2:6. doi: 10.1038/s43586-021-00092-5 37325194 PMC7614635

[69] de Melo LGP Nunes SOV Anderson G Vargas HO Barbosa DS Galecki P . Shared metabolic and immune-inflammatory, oxidative and nitrosative stress pathways in the metabolic syndrome and mood disorders. Prog Neuro-Psychopharmacol Biol Psychiatry. (2017) 78:34–50. doi: 10.1016/j.pnpbp.2017.04.027 28438472

[70] Yao J Zhu C Sun Y Huang Y Li Q Liao H . Insulin resistance: The role in comorbid type 2 diabetes mellitus and depression. Neurosci Biobehav Rev. (2025) 175:106218. doi: 10.1016/j.neubiorev.2025.106218 40403856

[71] Campbell IH Frye MA Campbell H . Metabolic plasticity: an evolutionary perspective on metabolic and circadian dysregulation in bipolar disorder. Mol Psychiatry. (2025) 30:5600–12. doi: 10.1038/s41380-025-03123-9 40681844 PMC12532716

[72] Törmälehto S Svirskis T Partonen T Isometsä E Pirkola S Virtanen M . Seasonal effects on hospitalizations due to mood and psychotic disorders: a nationwide 31-year register study. Clin Epidemiol. (2022) 14:1177–91. doi: 10.2147/CLEP.S372341 36304786 PMC9595069

[73] Goldschmied JR Palermo E Sperry S Burgess HJ McCarthy M Yocum A . Seasonal variation in mood among individuals with and without bipolar disorder. J Affect Disord. (2025) 369:1131–5. doi: 10.1016/j.jad.2024.10.101 39447967 PMC11608134

[74] Grootens KP Meijer A Hartong EG Doornbos B Bakker PR Al Hadithy A . Weight changes associated with antiepileptic mood stabilizers in the treatment of bipolar disorder. Eur J Clin Pharmacol. (2018) 74:1485–9. doi: 10.1007/s00228-018-2517-2 30083876

[75] McElroy SL Keck PE . Metabolic syndrome in bipolar disorder. J Clin Psychiatry. (2014) 75:46–61. doi: 10.4088/JCP.13r08634 24502861

[76] Alonso‐Pedrero L Bes‐Rastrollo M Marti A . Effects of antidepressant and antipsychotic use on weight gain: a systematic review. Obes Rev. (2019) 20:1680–90. doi: 10.1111/obr.12934 31524318

[77] Gartlehner G Nussbaumer-Streit B Gaynes BN Forneris CA Morgan LC Greenblatt A . Second-generation antidepressants for preventing seasonal affective disorder in adults. Cochrane Database Systematic Rev. (2019) 2019:CD011268. doi: 10.1002/14651858.CD011268.pub3 30883669 PMC6422318

[78] Seiter HT Fellendorf FT Popkova D Reininghaus EZ . Weight gain under antipsychotic and mood stabilizing treatment: a narrative review about mechanisms and future options. Int J Bipolar Disord. (2025) 13:33. doi: 10.1186/s40345-025-00397-4 41288798 PMC12647513

[79] Greil W de Bardeci M Müller-Oerlinghausen B Nievergelt N Stassen H Hasler G . Controversies regarding lithium-associated weight gain: case–control study of real-world drug safety data. Int J Bipolar Disord. (2023) 11:34. doi: 10.1186/s40345-023-00313-8 37840048 PMC10577117

[80] Gomes-da-Costa S Marx W Corponi F Anmella G Murru A Pons-Cabrera MT . Lithium therapy and weight change in people with bipolar disorder: a systematic review and meta-analysis. Neurosci Biobehav Rev. (2022) 134:104266. doi: 10.1016/j.neubiorev.2021.07.011 34265322

[81] Budin AJ Brown WA MacCormick AD Caterson I Sumithran P . Depressive symptoms at short‐, medium‐, and long‐term follow‐up after bariatric surgical procedures: a systematic review and meta‐analysis. Obes Rev. (2025) 26:e13927. doi: 10.1111/obr.13927 40222815 PMC12246895

[82] Fu R Zhang Y Yu K Mao D Su H . Bariatric surgery alleviates depression in obese patients: a systematic review and meta-analysis. Obes Res Clin Pract. (2022) 16:10–6. doi: 10.1016/j.orcp.2021.11.002 34802982

[83] van Andel E Vogel SWN Bijlenga D Kalsbeek A Beekman ATF Kooij JJS . Effects of chronotherapeutic interventions in adults with ADHD and delayed sleep phase syndrome (DSPS) on regulation of appetite and glucose metabolism. J Atten Disord. (2024) 28:1653–67. doi: 10.1177/10870547241285160 39318134

[84] Hammad M Shakoor K . Circadian rhythm modulation in metabolic disease: targeting clock genes for obesity and insulin resistance. Explor Endocrine Metab Dis. (2026) 3. doi: 10.37349/eemd.2026.101467

[85] Civelek E Ozturk Civelek D Akyel YK Kaleli Durman D Okyar A . Circadian dysfunction in adipose tissue: chronotherapy in metabolic diseases. Biol (Basel). (2023) 12:1077. doi: 10.3390/biology12081077 37626963 PMC10452180

[86] Bond DJ Kunz M Torres IJ Lam RW Yatham LN . The association of weight gain with mood symptoms and functional outcomes following a first manic episode: prospective 12‐month data from the Systematic Treatment Optimization Program for Early Mania (STOP‐EM). Bipolar Disord. (2010) 12:616–26. doi: 10.1111/j.1399-5618.2010.00855.x 20868460

[87] Neumeister A Praschak-Rieder N Hesselmann B Vitouch O Rauh M Barocka A . Rapid tryptophan depletion in drug-free depressed patients with seasonal affective disorder. Am J Psychiatry. (1997) 154:1153–5. doi: 10.1176/ajp.154.8.1153 9247407

